# A Fatty Acid Mouth Rinse Decreases Self-Reported Hunger and Increases Self-Reported Fullness in Healthy Australian Adults: A Randomized Cross-Over Trial

**DOI:** 10.3390/nu12030678

**Published:** 2020-03-02

**Authors:** Andrew Costanzo, Catherine G. Russell, Simone Lewin, Russell Keast

**Affiliations:** CASS Food Research Centre, School of Exercise and Nutrition Sciences Deakin University, 1 Gheringhap St, 3220 Geelong, VIC, Australia; andrew.costanzo@deakin.edu.au (A.C.); georgie.russell@deakin.edu.au (C.G.R.); simone.lewin@deakin.edu.au (S.L.)

**Keywords:** fat taste, satiety, mouth rinse, appetite, sensitivity

## Abstract

Fatty acid (FA) chemoreception in the oral cavity, known as fat taste, may trigger a satiety response that is homologous to FA chemoreception in the gastrointestinal tract. In addition, individuals with an impaired fat taste sensitivity are more likely to have an impaired satiety response. This study aimed to assess the effect of an FA mouth rinse on self-reported appetite, and to determine if the effect is modified by fat taste sensitivity. Thirty-one participants (age, 32.0 ± 8.4 y; body mass index (BMI), 26.1 ± 8.1 kg/m^2^) were studied on four separate days to evaluate the effect of a 20 mM oleic acid (OA) mouth rinse (in duplicate) compared to a control (in duplicate) on self-reported appetite by using a visual analogue scale (VAS) every 30 min for three hours following a standardized low-fat breakfast. The area under the curve ratings for fullness were greater (*p* = 0.003), and those for hunger were lower (*p* = 0.002) following the OA rinse compared to the control. The effect of the OA rinse was greater in individuals who were hypersensitive to fat taste compared to moderately sensitive and hyposensitive individuals for fullness (*p* < 0.010) and hunger (*p* < 0.010) ratings. In summary, an OA mouth rinse decreases self-reported hunger and increases self-reported fullness, particularly in those who are more sensitive to fat taste. FA receptors in the oral cavity may be potential targets to regulate appetite.

## 1. Introduction

The chemoreception of fatty acid (FA) via FA receptors in the gastrointestinal tract (GIT) triggers satiety responses [1], specifically triggering the release of satiety hormones [2,3] and the slowing of gastric emptying [4,5]. This signaling pathway exists to reduce subsequent appetite throughout and following an eating event [2]. While most dietary fat is consumed in the form of triglyceride, triglycerides are hydrolyzed to FA by gastric lipases in the GIT [6] and, to a smaller degree, by salivary lipases in the oral cavity [7]. However, the consumption of free fatty acid (FFA) has a greater effect on appetite than the equicaloric consumption of triglyceride, likely because there is greater potential for FFA to bind to FA receptors without relying on gastric and salivary lipase activity [5,8].

A recent study by our research group identified that homologous FA receptors on enteroendocrine cells in the GIT were also housed within human taste papillae in the oral cavity [9], leading to the belief that the entire alimentary canal has a role in regulating food intake via nutrient sensing [10]. Namely, FA receptors cluster of differentiation (CD) 36, free fatty acid receptor (FFAR) 4, FFAR2, G protein-coupled receptor (GPR) 84 and potassium voltage-gated channel (KCNA) 2 were identified in human fungiform papillae taste bud cells (TBCs) [9]. CD36 has also previously been identified in human foliate and circumvallate papillae TBCs [11]. Knowing that these FA receptors are the same and may have similar function to the GIT, there is potential that oral exposure to FFA might also elicit a similar satiety response. The chemoreception of FA in the oral cavity, known as fat taste, is associated with the regulation of dietary fat and energy intake [12]. Therefore, we propose the idea of exposing FFA to the oral cavity to trigger a satiety response that reducing subsequent appetite.

One matter that should be noted is the large degree of variation in fat taste sensitivity amongst individuals, which is regulated by habitual dietary fat intake [13,14,15] and genetics [7,16,17]. Individuals with an impaired fat taste sensitivity require higher concentrations of FA in a food before eliciting a taste response [12] and are therefore more likely to (i) have a reduced satiety response following the consumption of fatty foods [18], (ii) consume greater quantities of fatty food [19], and (iii) be overweight or obese [1,20,21,22,23,24]. Therefore, it is possible that individuals with impaired fat taste might be less susceptible to a satiety response following a mouth rinse with FA.

The aim of this study was to assess whether oral exposure to a milk-based FA mouth rinse would affect self-reported appetite in healthy Australian adults. In addition, this study aimed to determine whether fat taste sensitivity would modify the aforementioned effect. We hypothesized that the FA mouth rinse would result in a suppressed feeling of appetite compared to the control mouth rinse, and that this effect would be more pronounced in individuals who had a greater fat taste sensitivity.

## 2. Materials and Methods

### 2.1. Participants

**Figure 1:**
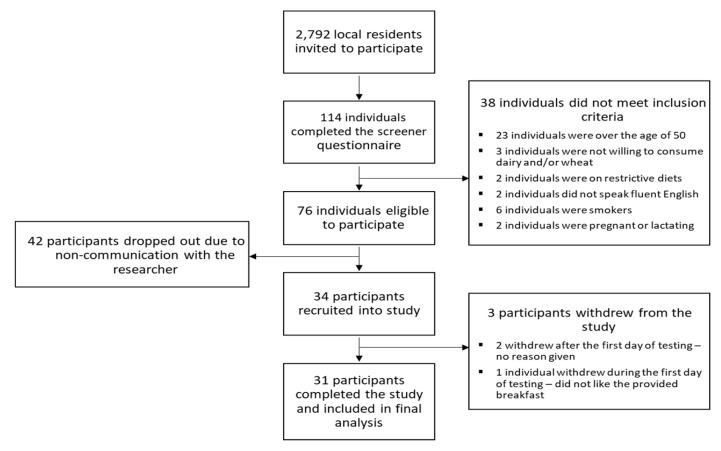
Consort diagram of participant recruitment, disposition status and analysis.

Residents from the Burwood (Melbourne, VIC, Australia) area near the Deakin University Burwood campus who had previous signed up to the CASS Food Research Centre Consumer Database (*n* = 2792) were invited via email to participate in this study. The invitation email contained a screener questionnaire to determine eligibility. Individuals were eligible to participate if they were aged between 18 and 50 years, were able to attend four laboratory testing sessions in the CASS lab at Deakin University, were willing to fast overnight prior to each testing session, and were willing to consume milk and wheat products for breakfast. Individuals were excluded from recruitment if they were allergic or intolerant to wheat or dairy, were smokers, were non-fluent in English, were pregnant or lactating, were on a diet that restricted energy consumption, and/or had an illness or condition that precluded them from fasting overnight (e.g., diabetes mellitus). One hundred and fourteen individuals completed the screener questionnaire, 76 of them met eligibility criteria, 42 dropped out due to non-communication with the researcher, and 34 were recruited into the study (Figure 1).

All subjects gave their informed consent for inclusion before they participated in the study. The study was conducted in accordance with the Declaration of Helsinki, and the protocol was approved by the Deakin University Faculty of Health Human Ethics Advisory Group (HEAG-H 182_2018). This trial was registered as a clinical trial with the Australian New Zealand Clinical Trials Registry (ID: ACTRN12619000668101).

### 2.2. Study Outline

**Figure 2:**
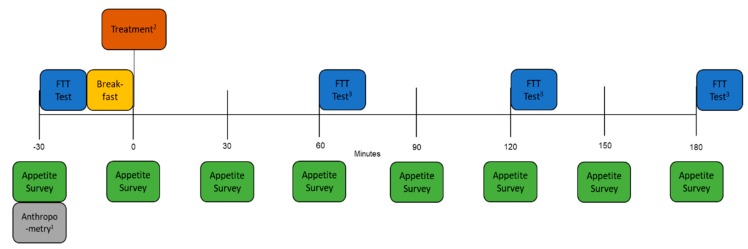
Outline of study design. FTT: fat taste threshold; ^1^ Anthropometry was only measured during the first day of testing; ^2^ Treatment was either the oleic acid (OA) or control mouth rinse, which were randomly allocated to each participant; ^3^ Subsequent FTT tests after the first were only measured on the first two days of testing.

Participants attended the CASS lab for data collection on four separate days. Each day began at 8:30 a.m. An outline of the study design is shown in Figure 2. Upon arrival to the CASS lab, participants verbally confirmed that they had not eaten or drank anything other than water in the last 10 h. On day 1, anthropometric measures were collected in order to calculate breakfast quantity. Following that, or at the beginning of all other days, participants completed an appetite questionnaire to determine their baseline appetite. Next, participants were assessed for their taste sensitivity to oleic acid (OA) by using a fat taste threshold (FTT) test. Immediately after the FTT measure was completed, participants were provided with a fat-free breakfast to consume within a 15 min period. At 9 a.m., the treatment, which was a 30 s mouth rinse of a solution that contained skim milk with the addition of either 20 mM OA or no added FA (control), was provided. Immediately following the treatment, participants completed another appetite questionnaire. At this point, participants were free to leave the lab. During this time, university staff or students were asked to return to their offices or the library during this period, and participants who were not associated with the university were asked to wait in a reserved waiting room in order to reduce the non-laboratory environmental variables. Participants were instructed to complete an online appetite questionnaire every 30 min (9:30 a.m., 10:00 a.m., 10:30 a.m., etc.) and to refrain from eating or drinking anything other than water for the next 3 h (until completion of the 12 p.m. questionnaire). A text message was sent to each participant’s mobile device every 30 min with a link to the CASS Online Portal to remind them to complete the questionnaire at the appropriate times and to continue to refrain from eating and drinking.

The study was a cross-over design, so the OA treatment was provided on two of the days and the control was provided on the other two days for each participant. The order of treatment was determined by creating a master list of random treatment orders by using Excel software functions = RAND and = IF (Microsoft Office 365 ProPlus, Redmond, WA, USA) prior to recruitment. Upon the recruitment of each participant, they were allocated to a treatment order in this list from top to bottom. Participants were not aware of which treatments they were being provided or their overall treatment order. Researchers were not blinded to which treatments were being provided to participants. There was a washout period of 24 h based on evidence that acute fat taste perception returns to pre-fasted levels after an 11 h fast in rodents [25]. Therefore, all participants were asked to fast overnight (at least 10 h) to standardize appetite and taste perception.

On days 1 and 2 only, participants were also asked to return to the CASS lab on every hour (10 a.m., 11 a.m., 12 p.m.) for additional FTT measures.

### 2.3. Appetite Questionnaire

Participants were asked to complete an appetite questionnaire every 30 min from the onset of each session (8:30 a.m. to 12:00 p.m.). The questionnaire was completed with the use of the Compusense Cloud computer software as part of the Compusense Academic Consortium (Compusense Inc., Guelph, Ontario, Canada). If participants were in the CASS lab when required to complete an appetite questionnaire, they were prompted to use the lab computers. Otherwise, they completed the questionnaire on their mobile device or personal computer by logging in to the CASS Online Portal. A questionnaire was only accepted if it was completed within 10 min of the scheduled time. For example, the 10:30 a.m. questionnaire would only be accepted if it was completed between 10:20 a.m. and 10:40 a.m.

The questionnaire contained 6 items on overall appetite [18] and 4 items on desire to eat foods of a specific taste [26] where participants rated their response on a 100 mm visual analogue scale (VAS). Items included “How full do you feel?” (fullness), “How hungry are you?” (hunger), “How strong is your desire to eat?” (desire), “How much food could you eat right now?” (quantity), “Do you feel you could eat a snack right now?” (snack), “Do you feel you could eat a full meal right now?” (meal), “Would you like to eat something sweet?” (sweet), “Would you like to eat something savory?” (savory), “Would you like to eat something fatty?” (fatty), and “Would you like to eat something salty?” (salty). All items were anchored at 0 and 100 mm, with respective labels of “Not full at all” and “Extremely full” or similar depending on the item (Image S1).

### 2.4. Fat Taste Threshold (FTT)

The detection threshold to OA was measured by using established methods [27]. Food-grade OA (Merck KGaA, Darmstadt, Germany) was stored under nitrogen gas below 4 °C. OA was added at varying concentrations (0.02, 0.06, 1.00, 1.40, 2.00, 2.80, 3.80, 5.00, 6.40, 8.00, 9.80, 12.00, and 20.00 mM) to long-life, fat-free milk (Devondale, Melbourne, VIC, Australia). All of the preparations were mixed with 5% (wt:vol) gum arabic (TIC Gums, Lane Cova, NSW, Australia) and 5% (vol:vol) liquid paraffin (Faulding Remedies, Melbourne, VIC, Australia) to produce perceptually identical textural attributes, including viscosity and lubricity between the OA and control samples. To prevent the oxidation of OA, all samples were mixed with 0.01% wt:vol Ethylenediaminetetraacetic acid (Merck KGaA, Darmstadt, Germany). Samples were homogenized at room temperature for 30 s/100 mL at 12,000 rpm (average centrifugal force: 3220× *g*) (Silverson L4RT homogenizer, East Longmeadow, MA, USA), prepared ≤2 h before testing, and served at room temperature. The control samples were prepared in the same way but without added OA. Participants were asked to rinse their mouths with water before beginning the task and between sample sets. To prevent confounding non-taste sensory inputs (e.g., being able to see or smell differences between samples), participants wore nose clips and all of the tests were conducted under red light.

FTT was determined by using an ascending series 3-alternate forced choice (3-AFC) methodology [27], which is a test to select a sample among a set of 3 that differs in a known attribute. To familiarize participants with the taste attribute of OA and to reduce sensory fatigue, participants were initially provided with warm-up sets before the 3-AFC test. A warm-up set contained an OA sample (initially, 3.8 mM) and a control sample. If participants were unable to perceive a difference between the control and OA sample during the first warm-up set, then they were provided with a new warm-up set at 8 mM.

The 3-AFC test began with the highest OA concentration sample set that could not be differentiated from the control during warm-up (i.e., the 3-AFC test began with 0.02 mM if the participant was able to differentiate the 3.8 mM sample from the control sample). Participants were provided with multiple sample sets, each containing 3 randomly ordered samples: 2 control samples and 1 sample containing OA. Participants were asked to taste each sample in the set and identify the sample that matched the taste quality from the warm-up sets. The correct identification of the OA sample resulted in the participants repeating the same sample set. The incorrect identification of the OA sample resulted in a new sample set with a higher concentration of OA. This continued in an ascending order from the initial concentration to the highest (20 mM) concentration. The endpoint was defined as the concentration of OA that was correctly identified in 3 consecutive sample sets of the same concentration, which was in line with commonly established sensory testing procedures [27]. Endpoints were transformed to an ordinal variable ranging from 0 to 12, with higher ranks implying a lower sensitivity to OA [19].

### 2.5. Breakfast

Following the initial FTT measure, participants were provided with a breakfast that consisted of WeetBix (Sanitarium, Berkeley Vale, NSW, Australia) and fat-free skim milk (Devondale, Melbourne, VIC, Australia) at a ratio of 26 g of WeetBix per 100 g of milk, based on the manufacturer’s serving recommendations. Participants had the option of having their milk served warm. Participants were required to consume the provided breakfast in full within a 15 min period.

The quantity of breakfast was served at 9% of the estimated energy requirement (EER) based on benchtop testing to determine a quantity that was comfortably filling but not overfilling. The EER was determined by calculating basal metabolic rate (BMR) by using the Schofield equation [28] multiplied by a self-reported physical activity level (PAL) to account for the energy expenditure of the individual. A list of validated PAL descriptions [29] was provided to the participants during the screener questionnaire so that they could estimate their baseline PAL. Participants were also asked if they regularly performed any additional strenuous leisure activities (30–60 min) throughout the week and, if so, how often. Additional strenuous leisure activities were added to the baseline PAL at 0.3 PAL units per day.

To calculate the BMR, body weight was measured after the removal of shoes, heavy clothing and any items in their pockets by using electronic scales (OHAUS NV4101, Parsippany-Troy Hills, New Jersey, USA), and height was measured by using a free-standing stadiometer (seca GmbH & Co. KG., Hamburg, Germany). The body mass index (BMI) was calculated as weight (kg)/height (m)^2^.

### 2.6. Treatment Mouth Rinses

The OA mouth rinse was prepared in the same way as the solutions for the FTT test, but at a 20 mM concentration of OA. The control mouth rinse was prepared similarly, but without OA.

At 9 a.m., participants were provided with 30 mL of either the OA or control mouth rinse and asked to swish the entire solution in their mouths for 30 s. Participants were instructed to ensure that they cover their entire oral cavity with the solution during swishing. Once 30 s was up, participants expelled the solution, rinsed their mouth with water once, and then completed an appetite questionnaire. Researchers visually confirmed that participants complied with the rinse for the full 30 s.

### 2.7. Statistical Analysis

All analyses were conducted by using SPSS (v25.0, IBM Corporation, Armonk, NY, USA). Numerical variables are reported as mean and standard deviation (SD). All variables were visually assessed for normality by using histograms with standard normality curves. The FTT was split into tertiles (hypersensitive, moderately sensitive and hyposensitive) to compare groups of individuals with different OA taste sensitivities. A one-way analysis of variance (ANOVA) was used to compare differences in descriptive variables between these three groups.

The effects of the OA mouth rinse on appetite ratings (fullness, hunger, desire, quantity, meal, snack, sweet, savory, fatty, and salty) were assessed by using linear mixed models including time (−30 min to 180 min), treatment (OA or control), time × treatment interaction, and the day of testing as fixed effects; the participant was used as a mixed effect to control for duplicate measures of each treatment. The same analysis was conducted to assess the effect of the OA mouth rinse on FTT. To assess FTT as an effect modifier of the OA mouth rinse on appetite, the area under the curve (AUC) was calculated manually as the sum of the areas within each trapezoid under the appetite rating vs. time curve (mm × h) [30]. Effect modifications on the appetite rating AUCs were assessed by using linear mixed models including treatment, FTT tertiles, treatment × FTT tertile interactions, and the day of testing as fixed effects; the participant was used as a mixed effect to control for duplicate measures of each treatment. Lastly, to assess the influence of ongoing FTT tests on the treatment effect, a linear mixed model was used that including days (days 1 and 2 and days 3 and 4), treatment, and days × treatment interactions as fixed effects; the participant was used as a mixed effect to control for duplicate measures of each treatment. Post-hoc Sidak’s tests were conducted for the above analyses, with *p*-values, standard error (SE) and 95% confidence intervals reported.

The number of participants recruited was initially based on a previous similar study [7], where 21 participants were sufficient to observe a significant difference (*p* < 0.05) in food intake and appetite perception following oleic acid and triglyceride (control) ingestion, with additional participants recruited to account for attrition. Following the completion of data collection, a retrospective sample size calculation was performed by using the G*Power software (v3.1, Institut für Experimentelle Psychologie, Düsseldorf, Germany) to determine if the current sample size had sufficient power. Based on the mean difference of the Hunger AUCs between the control (170 mm × h) and OA (147 mm × h) treatments with a pooled standard deviation of 30, it was determined that the study required a sample size of 27 for each treatment to achieve power of 80% and a level of significance of 5% (two-sided).

## 3. Results

Of the 34 participants who were recruited into the study, one withdrew during the first day of testing, while two participants withdrew from the study after the first day of testing (Figure 1). Thirty-one participants completed the study and were included in the final analysis. No harmful or unintended effects were observed following either of the mouth rinse treatments. No trial order effects were observed for any of the subsequent analyses.

The characteristic information of the participants is shown Table 1, both for the entire study sample and when stratified by the FTT tertile. There was a significant trend between the FTT tertiles and height, where shorter participants were more likely to have a reduced taste sensitivity to OA. The weight and BMI had similar trends between the FTT tertiles, where participants with a greater body mass were more likely to have a reduced taste sensitivity to OA, although these were not statistically significant.

### 3.1. The Effect of The OA Mouth Rinse on Appetite

To ensure that the ongoing hourly FTT tests (which were only conducted on days one and two of testing) did not influence appetite ratings, a comparison between the effects of the OA mouth rinse on days one and two and days three and four was conducted. There was no significant difference between the overall treatment effect sizes and the AUCs between these days of testing for all appetite measures (Table S1, Figure S1). Therefore, it was determined that the ongoing hourly FTT tests did not influence appetite ratings, and thus all days of testing were included in the below analyses.

**Figure 3:**
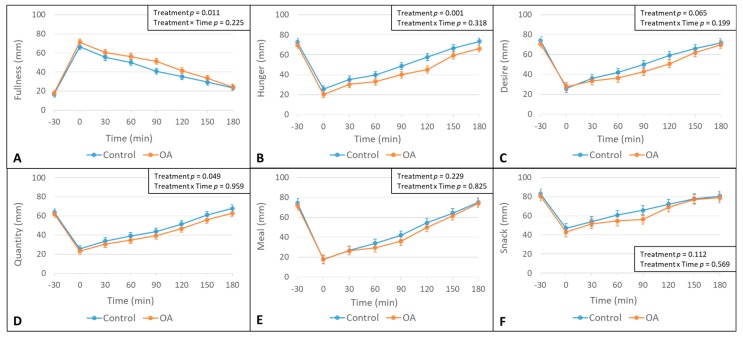
Means and SE (Standard Error) bars for self-reported appetite ratings in participants (*n* = 31) prior to and following mouth rinse treatments over time. Means, SE bars, and *p*-values were estimated by using a linear mixed model including treatment, time, and treatment × time interaction as fixed effects; the participant was used as a mixed effect to control for duplicate measures of each treatment (**A**): fullness ratings; (**B**): hunger ratings; (**C**): desire to eat ratings; (**D**): quantity of food that could be consumed ratings; (**E**): desire to eat a meal ratings; and (**F**): desire to eat a snack ratings.

**Figure 4:**
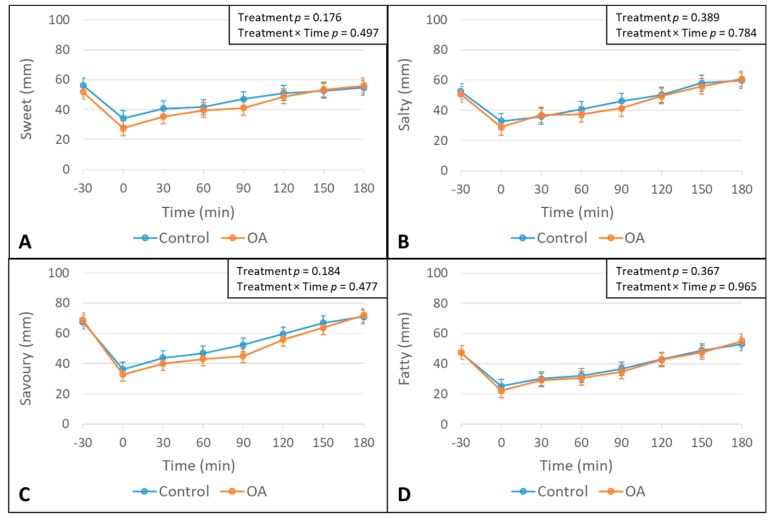
Means and SE bars for self-reported desire to eat foods with specific taste ratings in participants (*n* = 31) prior to and following mouth rinse treatments over time. Means, SE bars, and *p*-values were estimated by using a linear mixed model including treatment, time, and treatment × time interaction as fixed effects; the participant was used as a mixed effect to control for duplicate measures of each treatment. (**A**) Desire to Eat Sweet Food ratings; (**B**) Desire to eat Salty Food ratings; (**C**) desire to eat savory food ratings; and (**D**) desire to eat fatty food ratings.

There was a significant effect of the treatment on fullness, hunger and quantity ratings, where overall fullness ratings were greater and overall hunger and quantity ratings were lower following the OA treatment, as compared to the control treatment (Figure 3). However, no significant treatment × time interactions were observed for any of the appetite ratings (Figure 3) and desires to eat foods of a specific taste (Figure 4).

### 3.2. The Effect of the OA Mouth Rinse on FTT

**Figure 5:**
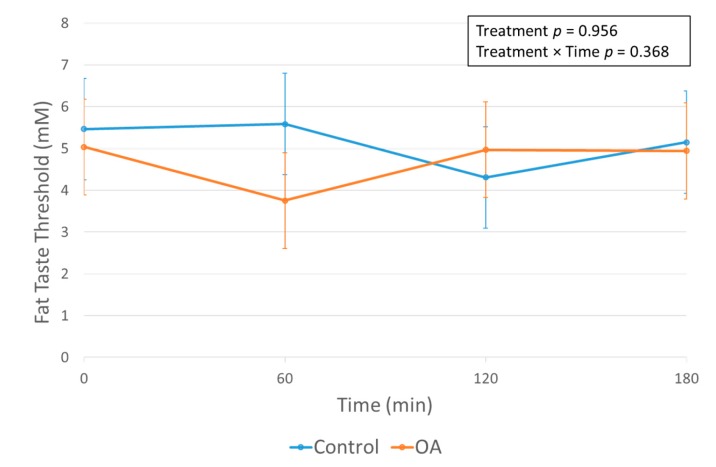
Means and SE bars for fat taste thresholds (FTT) in participants (*n* = 31) prior to and following mouth rinse treatments over time. Data were only collected during days 1 and 2 of testing. Means and SE bars, and *p*-values were estimated by using a linear mixed model including treatment, time, and treatment × time interaction as fixed effects; the participant was used as a mixed effect to control for duplicate measures of each treatment.

There was no significant effect of the OA mouth rinse on FTT (Figure 5), suggesting that fat taste sensitivity remains relatively stable in the hours following acute exposure to FA.

### 3.3. The Influence of FTT (Effect Modification) on the Efficacy of the OA Mouth Rinse on Appetite

FTT was observed to modify the effect of the OA mouth rinse on self-reported fullness and hunger (Table 2), where the effect was greater in hypersensitive individuals and lesser in hyposensitive individuals. While there was no significant effect of FTT on the other measures of appetite, the AUCs for desire, quantity, meal and sweet had similar trends in the level of effect between the FTT tertiles, where the effect was greatest in hypersensitive individuals.

## 4. Discussion

The current study aimed to assess whether oral exposure to a milk-based FA mouth rinse would affect self-reported appetite, and the results showed that some of the appetite ratings were affected. Specifically, the OA treatment increased fullness and reduced hunger compared to the control treatment. This suggests that oral FA exposure influences appetite by triggering the satiety cascade, potentially via the slowing of gastric emptying [4,5,8] and the transient stimulation of satiety hormones [31,32].

While no other study has assessed the effect of oral FA exposure on appetite, there has been previous research on the physiological response to sham-feeding fatty food or triglyceride. The sham-feeding or oral stimulation of dietary fat has been shown to increase plasma triglyceride concentrations [33,34], gastric lipase secretion [35], and the transient stimulation of cholecystokinin, pancreatic polypeptide and peptide YY [31,32]. This suggests that the role of the fat taste system is not solely to regulate dietary fat intake; its role is also to prepare the body for fat digestion and absorption.

The timing of the effect is also of interest, as there appeared to be some delay before the satiety effect became notable, suggesting that an FA mouth rinse program may be a consumer-friendly treatment to reducing snacking between meals or overall food intake throughout a meal if timed correctly. The effect size was relatively small, with the greatest difference in ratings between the OA and control treatments being 10.5 out of 100 mm on the hunger VAS. This translates to a 10.5% reduction in the overall feeling of hunger. For comparison, the consumption of a lunch meal should equate to a change in VAS ratings by about 55–65 mm [18]. Flint et al. [36] suggested that a 10 mm change on a VAS is meaningful in the context of appetite. Further research on the effect of a fatty acid mouth wash on actual food or energy intake might demonstrate a greater clinical significance of the treatment. In addition, ongoing OA mouth rinse treatments might have a larger cumulative influence on long-term energy intake and weight loss or maintenance.

The OA mouth rinse appeared to have the greatest effect on fullness, hunger, desire to eat, and desire to eat a snack around 90 min post-treatment, but it had negligible effect on desire to eat a meal or quantity of food that could be consumed. For the most part, the effect of the OA mouth rinse was temporary and returned to appetite ratings similar to the control after 150–180 min post-treatment, except for hunger. This suggests that the OA mouth rinse is more likely to be useful to suppress snacking between meals. However, this may be due to the timing of the treatment being immediately after a breakfast meal. These results should be interpreted as indicative only, as there was not enough power to determine if there was a true treatment x time interaction. More research on the timing of the treatment is needed to determine if it can influence a reduction in overall energy intake during and between meals. Moreover, the OA mouth rinse had no significant effect on desire to eat food of specific tastes. If the mechanism of the OA mouth rinse is a slowing of the gastric emptying and stimulation of satiety hormones, then we hypothesize that the mouth rinse should not have a major impact on desire of foods of any taste quality. The results from this study appear to confirm this.

Interestingly, there was no effect of the OA mouth rinse on subsequent FTT. This differs from previous reports of acute lipid exposure on the tongues of rodents causing an immediate reduction in protein levels of the FA receptor CD36 [25]. The difference may have been due to the dosage of the treatment in the current study, as the concentration of OA that was used was relatively low compared to pure triglyceride. Nonetheless, this is an opportune finding because it indicates that FTT testing does not interfere with the overall ability to perceive FA. Assessing the change in FTT in humans following a high triglyceride exposure or the consumption of a high-fat meal would be interesting for future research.

It should be noted that the results from the current study suggest that the OA mouth rinse was less effective in individuals who are hyposensitive to fat taste, likely to due to the attenuated fat taste-specific satiety response [18]. This may be problematic, as individuals who are hyposensitive are more likely to be overweight or obese, which are the population that would benefit most from a treatment that aids in appetite suppression [1,20,21,22,23,24]. Strategies to increase the effectiveness of the treatment on hyposensitive individuals might be to increase the concentration or duration of the FA exposure or to use unsaturated FA, which has a greater affinity for FA receptor activation [22], although these would need additional testing to confirm. In addition, previous works have shown that short-term low-fat dietary intake can increase fat taste sensitivity [13,14,15] via the upregulation of FA taste receptor gene expression [37]. Therefore, an appetite suppression treatment that uses an FA mouth rinse might work best in tandem with a low-fat diet. This might also aid individuals through the initial “difficult” phase of dieting and lead to a greater adherence to low-fat diets.

One limitation of the current study is that no information was collected on actual dietary intake or physiological satiety factors. Rather, the study relied on self-reported appetite ratings as a proxy for satiety and prospective consumption. Future studies will need to assess the same treatments on actual markers of dietary behavior, such as ad libitum buffet intake or transient satiety hormones, to determine whether an FA mouth rinse treatment may be a useful aid for appetite suppression. Second, the current study did not collect information on menstrual cycles, which may have impacted appetite between days of testing in females [38]. Third, the dietary information of the participants on the days prior to data collection was not measured. While participants did fast for at least 10 h prior to the breakfast, it is acknowledged that variation in energy intake within the last 24 h may affect appetite [39]. Fourth, while participants were asked to remain in office or other quiet spaces during the interim period between FTT tests on days one and two, we acknowledge that participants may not have adhered to this request. Variation in non-laboratory environment during this time may have influenced appetite. Fifth, the temperature of the breakfast was not standardized, which may have some influence on the satiety response following its consumption [40]. Lastly, the sample size was calculated to be sufficient to observe differences between the AUCs for appetite ratings between the OA and control treatments. However, the sample size may not have been sufficient for additional analyses such as treatment × time interactions.

## 5. Conclusions

The findings from the current study suggest that an FA mouth rinse may be a promising treatment to reduce short-term satiety to aid appetite control and weight management. However, ongoing research is necessary to determine the clinical significance of the treatment on actual dietary behavior and physiological markers, especially in overweight and obese individuals.

## Figures and Tables

**Table 1:** Characteristics of participants.

		FTT Tertiles			
	Overall	Hypersensitive	Moderately Sensitive	Hyposensitive	ANOVA *p*-Value
*n*	31	9	11	11	−
% female	55	46	58	60	0.432
Age (years)	32.0 (8.4)	32.0 (6.5)	30.8 (8.8)	33.2 (9.2)	0.385
Height (cm)	168.6 (8.6)	170.9 (7.8)	169.0 (8.8)	166.0 (8.1)	0.029
Weight (kg)	74.2 (23.0)	69.9 (11.3)	75.7 (26.1)	76.2 (26.1)	0.400
BMI (kg/m^2^)	26.1 (8.1)	23.9 (3.4)	26.5 (9.2)	27.6 (9.0)	0.100
FTT	3.7 (3.0)	1.4 (0.4)	2.4 (0.5)	7.0 (2.1)	< 0.001

Data are presented as mean (SD) for continuous variables. The ANOVA *p*-value represents the statistical trend in the variable between the FTT tertiles.

**Table 2:** Mean area under curves (AUCs) and differences in self-reported appetite in hypersensitive, moderately sensitive and hyposensitive individuals following mouth rinses.

	Control mm × hr (95% CI)	OA mm × hr (95% CI)	Difference mm × hr (95% CI)	FTT Tertile × Treatment Interaction (*p*)
**Fullness AUC**				<0.001
Overall	147 (128, 166)	168 (149, 187)	21 (7, 35) **	
Hypersensitive	139 (113, 165)	176 (150, 203)	37 (11, 63) **	
Moderately sensitive	149 (126, 173)	171 (146, 196)	22 (−4, 47)	
Hyposensitive	152 (124, 179)	156 (132, 181)	5 (−20, 30)	
**Hunger AUC**				0.028
Overall	170 (148, 192)	147 (125, 168)	−24 (−38, −9) **	
Hypersensitive	183 (154, 211)	140 (111, 169)	−42 (−69, −16) **	
Moderately sensitive	178 (153, 204)	148 (120, 175)	−31 (−57, −4)*	
Hyposensitive	150 (120, 180)	152 (125, 179)	2 (−24, 28)	
**Desire AUC**				0.095
Overall	173 (146, 200)	160 (133, 188)	−13 (−28, 3)	
Hypersensitive	181 (148, 215)	155 (120, 189)	−26 (−55, 2)	
Moderately sensitive	183 (152, 214)	168 (135, 200)	−15 (−44, 13)	
Hyposensitive	155 (120, 191)	159 (127, 191)	3 (−25, 31)	
**Quantity AUC**				0.133
Overall	157 (134, 181)	146 (123, 170)	−11 (−25, 3)	
Hypersensitive	172 (143, 201)	150 (120, 180)	−22 (−48, 4)	
Moderately sensitive	160 (133, 187)	145 (116, 173)	−15 (−41, 11)	
Hyposensitive	140 (109, 171)	144 (116, 172)	4 (−21, 29)	
**Meal AUC**				0.196
Overall	154 (126, 181)	147 (120, 175)	−6 (−23, 10)	
Hypersensitive	173 (138, 207)	150 (115, 185)	−23 (−53, 8)	
Moderately sensitive	154 (123, 186)	147 (114, 180)	−8 (−37, 22)	
Hyposensitive	134 (98, 170)	146 (113, 178)	11 (−18, 41)	
**Snack AUC**				0.389
Overall	227 (194, 260)	216 (183, 249)	−11 (−28, 7)	
Hypersensitive	229 (190, 269)	218 (178, 259)	−11 (−43, 22)	
Moderately sensitive	240 (203, 277)	223 (185, 262)	−17 (−49, 15)	
Hyposensitive	211 (169, 252)	206 (167, 244)	−5 (−36, 27)	
**Sweet AUC**				0.662
Overall	158 (126, 190)	151 (119, 183)	−7 (−23, 9)	
Hypersensitive	173 (134, 211)	150 (111, 188)	−23 (−53, 7)	
Moderately sensitive	172 (136, 208)	168 (131, 205)	−4 (−33, 26)	
Hyposensitive	130 (90, 169)	135 (98, 172)	6 (−24, 35)	
**Salty AUC**				0.098
Overall	157 (123, 191)	152 (118, 187)	−5 (−21, 11)	
Hypersensitive	162 (122, 202)	153 (112, 193)	−9 (−39, 21)	
Moderately sensitive	165 (127, 202)	148 (109, 187)	−17 (−46, 12)	
Hyposensitive	145 (103, 186)	156 (117, 195)	11 (−17, 40)	
**Savory AUC**				0.524
Overall	184 (155, 214)	175 (145, 205)	−9 (−26, 7)	
Hypersensitive	193 (157, 229)	178 (141, 215)	−15 (−45, 16)	
Moderately sensitive	193 (159, 227)	173 (138, 208)	−20 (−50, 10)	
Hyposensitive	167 (129, 205)	174 (140, 209)	7 (−22, 37)	
**Fatty AUC**				0.067
Overall	131 (102, 159)	129 (101, 158)	−1 (−15, 12)	
Hypersensitive	141 (108, 174)	135 (102, 169)	−6 (−31, 20)	
Moderately sensitive	140 (109, 171)	133 (100, 165)	−7 (−32, 18)	
Hyposensitive	111 (76, 145)	120 (88, 153)	9 (−15, 34)	

Difference between treatments calculated as OA—Control. Means (95% CIs) and *p*-values were estimated by using a linear mixed model including FTT tertile, treatment, and FTT tertile × treatment interactions as fixed effects; the participant was used as a mixed effect to control for duplicate measures of each treatment. Post hoc Sidak’s test and CIs are reported. * *p* < 0.05, ** *p* < 0.01.

## Supplementary Materials

The following are available online at https://www.mdpi.com/2072-6643/12/3/678/s1, Image S1: Appetite Questionnaire, Table S1: Comparison of appetite rating AUCs between days one and two and days three and four, Figure S1. Means and 95% CIs for self-reported appetite ratings in participants (*n* = 31) on days one and two (ongoing FTT tests) and days three and four (one initial FTT test) prior to and following mouth rinse treatments over time.
