# Supplementary material for: A Fatty Acid Mouth Rinse Decreases Self-Reported Hunger and Increases Self-Reported Fullness in Healthy Australian Adults: A Randomized Cross-Over Trial
Source: Nutrients. 2020 Mar 2;12(3):678. doi: 10.3390/nu12030678 (PMC7146224; doi:10.3390/nu12030678)
Supplement: Supplementary file 1 [file nutrients-12-00678-s001.zip › Image S1.pdf]

Oral Rinse Study Satiety Questionnaire.

For this study, we ask that you complete a Satiety Questionnaire every 30 minutes from the onset of each session. Please answer the questions as accurately as possible. You will be notified via SMS when you need to complete the next questionnaire. Please complete these questionnaires promptly to allow as much time as possible between each questionnaire.

This questionnaire should take you approximately **2 minutes** to complete.

PRESS THE 'NEXT' BUTTON TO BEGIN

SATIETY QUESTIONNAIRE

Instructions: For each question, please rate your answer on the scale as accurately as possible. Please complete this questionnaire promptly.

How full do you feel?

Not full at all — Extremely full

How hungry are you?

Not hungry at all — Extremely hungry

How strong is your desire to eat?

Strong desire not to eat — Strong desire to eat

How much food could you eat right now?

Nothing at all — The most I have ever eaten

Do you feel you could eat a snack right now?

I could not eat a snack right now — I could eat a snack right now

Do you feel you could eat a full meal right now?

I could not eat a full meal right now — I could eat a full meal right now

Would you like to eat something sweet right now?

Not at all — Yes, very much

Would you like to eat something savoury right now?

Not at all — Yes, very much

Would you like to eat something fatty right now?

Not at all — Yes, very much

Would you like to eat something salty right now?

Not at all — Yes, very much

FINISHED!

PRESS THE 'FINISH' BUTTON TO SUBMIT YOUR RESULTS

Reminder, you will complete this questionnaire every 30 minutes throughout this session. Please ensure you complete the next questionnaire promptly when notified.
If you have any questions or concerns, please return to the CASS lab (J1.05) and ring the doorbell to get the attention of the researcher.

Finished
