# Supplementary material for: A Fatty Acid Mouth Rinse Decreases Self-Reported Hunger and Increases Self-Reported Fullness in Healthy Australian Adults: A Randomized Cross-Over Trial
Source: Nutrients. 2020 Mar 2;12(3):678. doi: 10.3390/nu12030678 (PMC7146224; doi:10.3390/nu12030678)
Supplement: Supplementary file 1 [file nutrients-12-00678-s001.zip › Table S1 and Figure S1.pdf]

Table S1 – Comparison of appetite rating AUCs between days 1 & 2 and days 3 & 4.

	Days 1 & 2 mm × hr (95% CI)	Days 3 & 4 mm × hr (95% CI)	Difference (95% CI)	Days (p)	Days × Treatment (p)
Fullness	158 (140, 177)	156 (137, 175)	-2.43 (-16.0, 11.1)	0.723	0.198
Hunger	156 (135, 178)	162 (141, 184)	6.23 (-8.00, 20.4)	0.385	0.662
Desire	161 (134, 188)	174 (147, 201)	13.5 (-1.82, 28.9)	0.083	0.690
Quantity	145 (122, 169)	159 (136, 182)	13.6 (-0.27, 27.5)	0.055	0.850
Meal	143 (115, 171)	158 (131, 186)	15.3 (-0.61, 31.1)	0.059	0.672
Snack	215 (182, 249)	228 (195, 261)	12.7 (-4.49, 29.9)	0.146	0.942

Difference between days calculated as 3&4 – 1&2. Means (95% CIs) and p-values were estimated using a linear mixed model including days, treatment, and days × treatment interactions as fixed effects, and participant as a mixed effect to control for duplicate measures of each treatment.

Figure S1. Means and 95% CIs for self-reported appetite ratings in participants (n = 31) on days 1 & 2 (ongoing FTT tests) and days 3 & 4 (one initial FTT test) prior to and following mouth rinse treatments over time.

Means and 95% CIs were estimated using a linear mixed model including days, time, and days × time interaction as fixed effects, and participant as a mixed effect to control for duplicate measures of each treatment. A: Fullness ratings; B: Hunger ratings; C: Desire to Eat ratings; D: Quantity of Food that could be Consumed ratings; E: Desire to Eat a Meal ratings; F: Desire to Eat a Snack ratings.

A

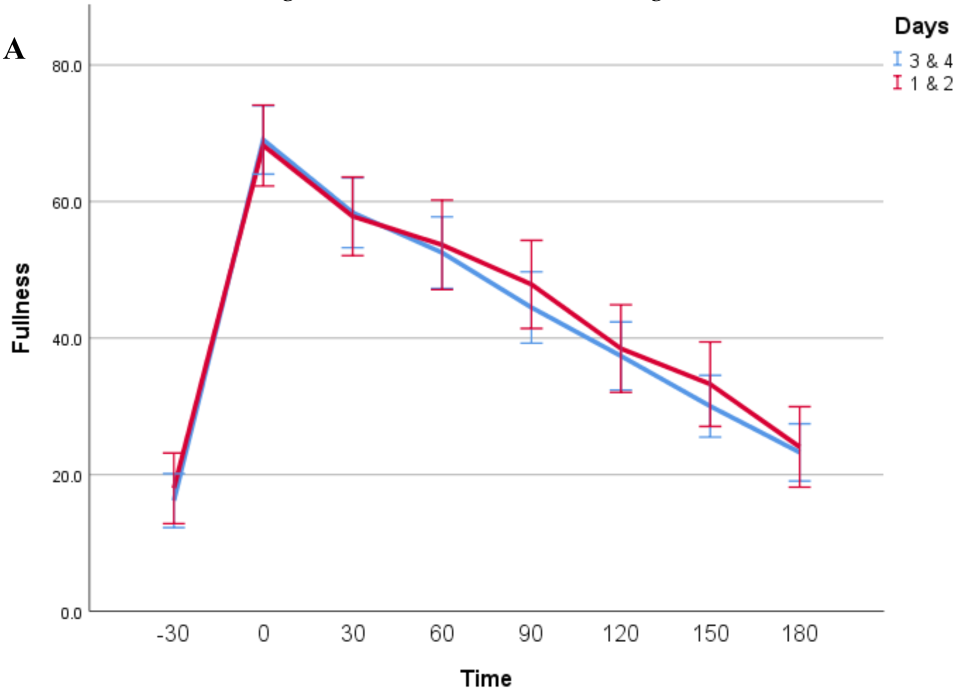

B

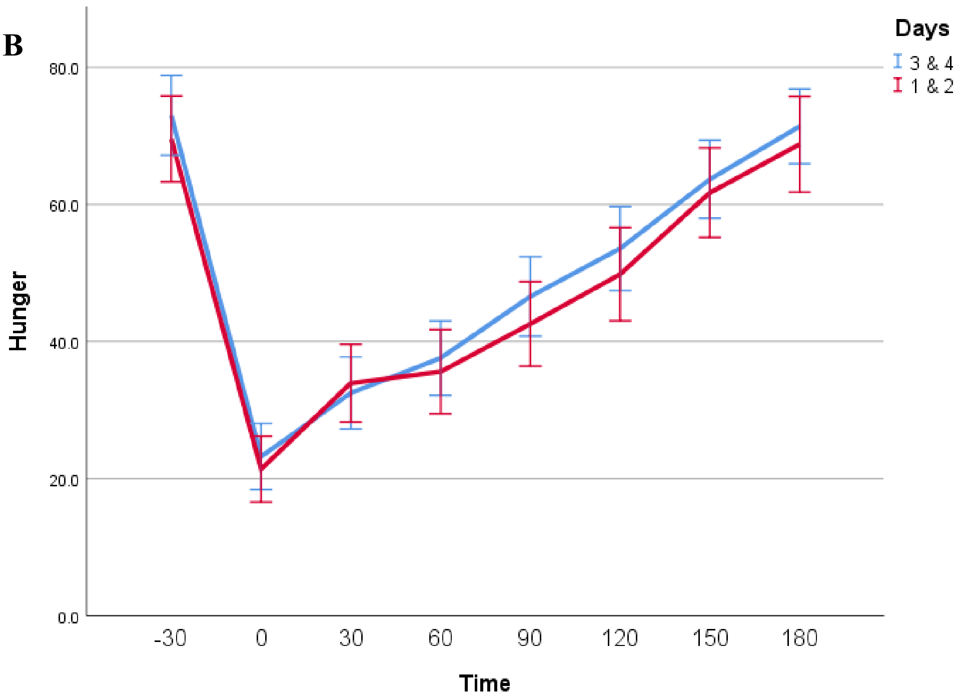
Days
3 & 4
1 & 2
Hunger
80.0
60.0
40.0
20.0
0.0
-30
0
30
60
90
120
150
180
Time

C

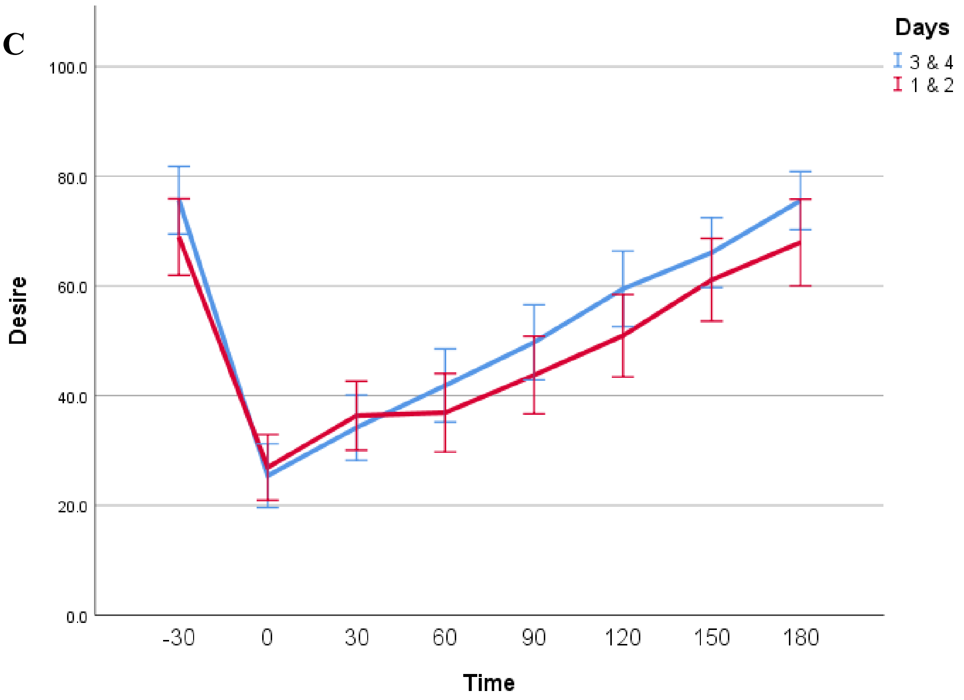
Days
3 & 4
1 & 2
Desire
100.0
80.0
60.0
40.0
20.0
0.0
-30
0
30
60
90
120
150
180
Time

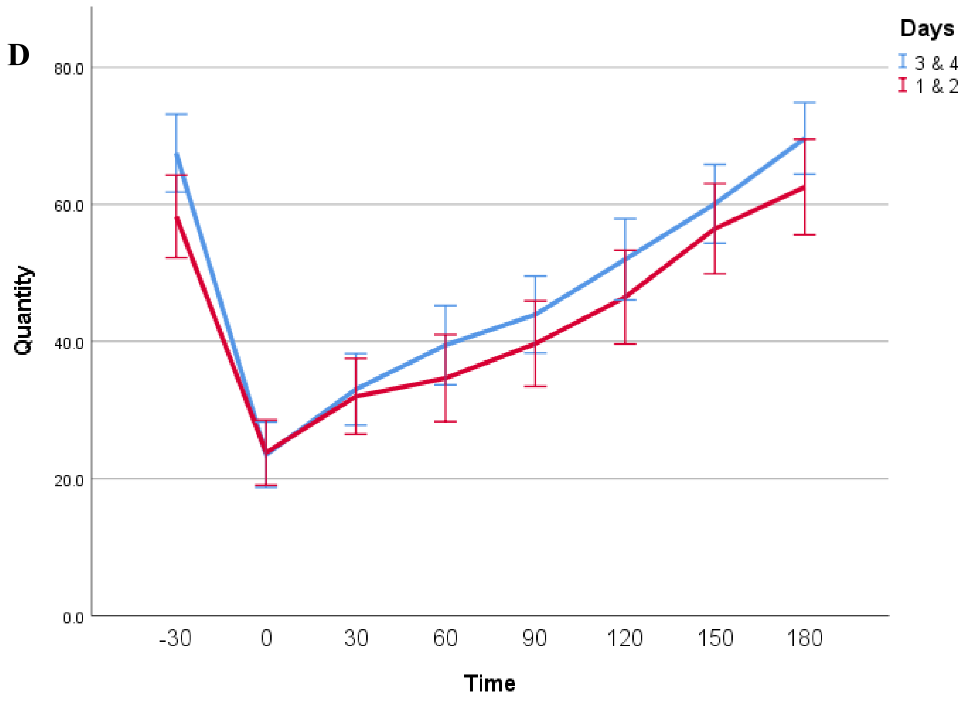
D
Quantity
80.0
60.0
40.0
20.0
0.0
-30
0
30
60
90
120
150
180
Time
Days
3 & 4
1 & 2

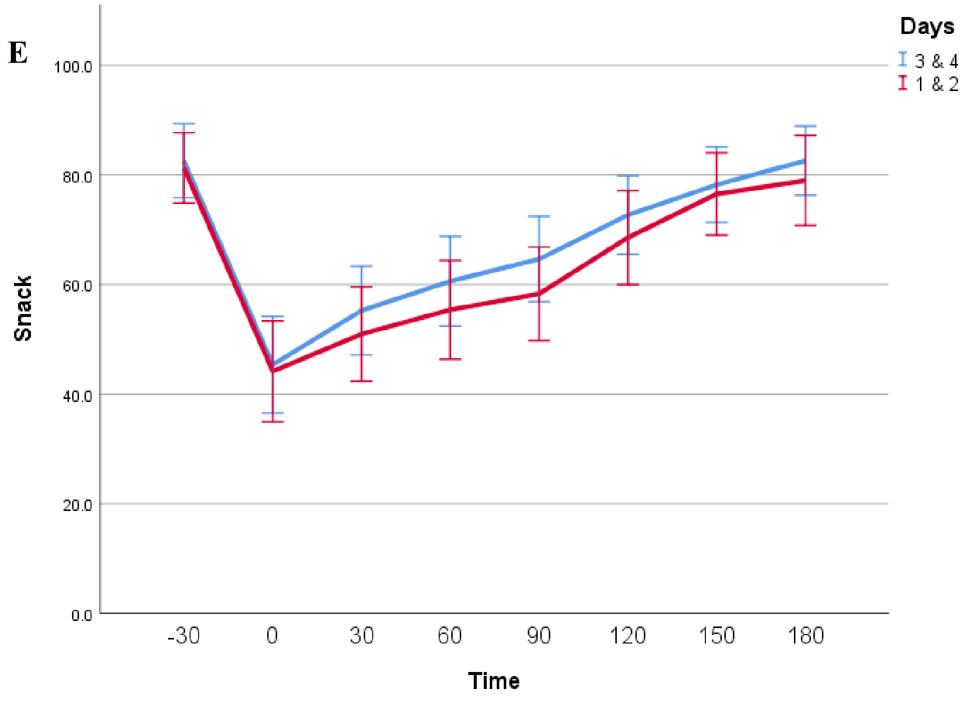
E
Snack
100.0
80.0
60.0
40.0
20.0
0.0
-30
0
30
60
90
120
150
180
Time
Days
3 & 4
1 & 2

F

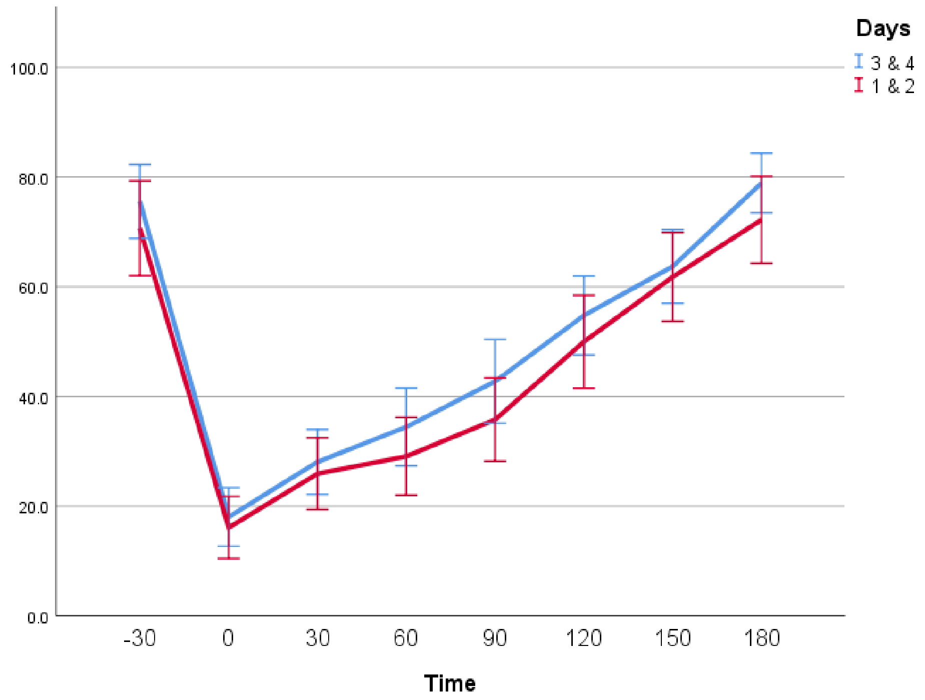
Days
3 & 4
1 & 2
100.0
80.0
60.0
40.0
20.0
0.0
Meal
-30
0
30
60
90
120
150
180
Time
